# Editorial: Enhancing gait therapy with artificial intelligence: current trends and future prospects

**DOI:** 10.3389/frobt.2026.1953216

**Published:** 2026-09-22

**Authors:** Ankit Vijayvargiya, Rajesh Kumar, Pilani Nkomozepi, Kieran Moran, Chandra Prakash

**Affiliations:** 1 School of Technology, Dhirubhai Ambani University, Gandhinagar, India; 2 Department of Electrical Engineering, Malaviya National Institute of Technology, Jaipur, India; 3 Department of Human Anatomy and Physiology, University of Johannesburg, Johannesburg, South Africa; 4 Department of Sport Science and Nutrition, Maynooth University, Maynooth, Ireland; 5 Department of Artificial Intelligence and Data Engineering, Malaviya National Institute of Technology, Jaipur, India

**Keywords:** artificial intelligence, digital biomarkers, gait analysis, movement disorders, orthopaedic surgery planning, rehabilitation, wearable sensors

## Introduction

Impaired mobility is among the most common impairments caused by neurologic, neurodegenerative, and orthopaedic disorders. Depending upon the cause being stroke, Parkinson’s disease, spinocerebellar ataxia, or osteoarthritis needing arthroplasty, the disruption in gait and mobility causes dependency, increased risk of falls, and decreased quality of life. Conventional methods used for assessing these disruptions have been based on visual inspection, rating scales, and occasional visits to clinics, which are time-consuming, observer-dependent, and has limited capacity of providing information on how a patient walks during daily routine activities or on the positioning of interventions and implants.

The current Research Topic was compiled to highlight how digital health technologies, such as wearable inertial sensors, markerless motion capture, exergame technology, and artificial intelligence/machine learning (AI/ML), can be employed to make movement assessment more objective, continuous, and personalizable. Five papers included in the current Research Topic present the research that covers a broad spectrum of clinical and technical Research Topic, from a randomized controlled trial of motor-cognitive exergame training in post-stroke rehabilitation to a narrative review of AI-assisted preoperative planning for total hip arthroplasty and three studies demonstrating the validation and application of sensor and machine-learning-based gait analysis in healthy subjects, patients with Parkinson’s disease and spinocerebellar ataxia.

## Overview of contributions

### Personalized motor-cognitive exergaming after stroke

Huber et al. present the results of the PEMOCS trial, a single-blind, randomized, controlled trial comparing the effects of conceptually guided, personalized, motor-cognitive exergame training with usual care in 46 community-living stroke survivors with chronic deficits. Although the primary outcome measure (global cognition assessed using the Montreal Cognitive Assessment) was not significantly different between the exergame and usual care groups, it was found that there were more responders in the exergame group. There were some interaction effects between time points in favour of the exergame group, for perceived mobility, intrinsic visual attention, working memory, outdoor gait speed and swing width. It is discussed how the frequency of training, exercise intensity, and a high-functioning baseline sample may have prevented the researchers from finding a bigger effect, and possible directions for future exergame-based interventions in chronic stroke patients are proposed.

### AI-assisted preoperative planning in total hip arthroplasty

Wang et al. present a narrative review of artificial intelligence-assisted, three-dimensional preoperative planning of the position and sizing of acetabular cups using CT scans in total hip arthroplasty. This review summarizes findings showing that AI-driven planning based on automated segmentation, deep learning landmark detection, and biomechanical simulation allows for a reduction in the mean angular error of cup inclination and anteversion to less than 3°, 80%–85% size-matching accuracy within one implant size, and at least 50% decrease in time needed for planning relative to manual templating. The authors also highlight important limitations of the existing evidence, including a lack of high-quality randomised studies, poor representation of revisions and complicated cases with acetabular defects among patients whose imaging scans were used to train the algorithms, and “black box” algorithms as important challenges impeding clinical adoption of this technology.

### A dual-depth, marker-less gait analysis system

Cafolla and Chaparro-Rico have performed system characterization of the current version of SANE (eaSy gAit aNalysis systEm), a real-time gait analysis system based on two depth cameras and pose estimation using artificial intelligence. Following the earlier clinical validation of SANE against marker-based methods, the authors concentrate on the robustness of this system in the present study. Over the course of four runs and 80 walks by a healthy individual during 1 week, with a re-test 1 week later, including system resets, the relative error for spatiotemporal parameters was mostly under 5%, and the standard deviation was on par with the results from previous studies with marker-based systems. The study illustrates that dual-camera processing could alleviate occlusions and allow for maintaining reliable, high-framerate (∼80 fps) real-time processing of gait data.

### Machine learning for Parkinson’s disease severity classification

Mittal et al. apply Decision Tree, Random Forest, XGBoost, and LightGBM classifiers to ground-reaction-force gait data to detect Parkinson’s disease and classify its severity on the Hoehn and Yahr scale, using two open-access benchmark datasets. They have achieved 98.25% accuracy on the PhysioNet gait dataset and 85% accuracy on another independent Figshare dataset, which is better than other classifiers and some earlier published works. Notably, the researchers use techniques of explainable artificial intelligence to show individual decision path that leads to every classification result, an effort aimed at building up clinical trust in the proposed model to be used as a quick, automatic test to screen Parkinson’s disease staging, while recognizing the necessity for future validations on wearable devices and prospective datasets.

### Clinic versus daily-life gait in spinocerebellar ataxia

Shah et al. compare the gait parameters derived using body-worn inertial sensors on the 2-min walk test done in a supervised environment, during an in-clinic appointment, and during unsupervised, day-to-day monitoring in a week in 26 patients with spinocerebellar ataxia (SCA types 1, 2, 3, and 6) and 13 age-matched healthy controls. Gait parameters derived during the in-clinic walk test were better at distinguishing the two groups. They had better correlations with clinical and self-reported parameters. Still, the two parameters of gait variability of stride timing, swing time variability and double-support time variability showed robust discrimination power in both cases. This research provides evidence for the complementary use of in-clinic and daily-life gait measurements: the former is preferred because of its higher sensitivity and clinical correlations used as the trial endpoint, whereas the latter is ecologically valid.

### Cross-cutting themes and future directions

Collectively, these studies reflect an increasingly important trend towards the use of AI and sensor technology for movement assessment to facilitate improved diagnosis, surgical planning, and rehabilitation. It is clear from these examples that objective digital metrics may be used to detect clinically significant alterations that may not be apparent by traditional methods, and stress the importance of studying motion both within the clinical setting and outside of it. Some common elements across all these studies include the need for explainable AI, reliable technology, and well-represented validation samples. The next step should be towards large-scale, diverse samples, as well as prospective multi-centre validation.

